# *Propionimicrobium lymphophilum* in urine of children with monosymptomatic nocturnal enuresis

**DOI:** 10.3389/fcimb.2024.1377992

**Published:** 2024-11-25

**Authors:** Naoto Nishizaki, Satoshi Oshiro, Mari Tohya, Shin Watanabe, Tadaharu Okazaki, Ken Takahashi, Teruo Kirikae, Toshiaki Shimizu

**Affiliations:** ^1^ Department of Pediatrics, Juntendo University Urayasu Hospital, Urayasu, Chiba, Japan; ^2^ AMR Research Laboratory, Juntendo Advanced Research Institute for Health Science, Juntendo University School of Medicine, Tokyo, Japan; ^3^ Division of Food Safety Information, National Institute of Health Sciences, Kawasaki, Kanagawa, Japan; ^4^ Department of Microbiome Research, Juntendo University Graduate School of Medicine, Tokyo, Japan; ^5^ Department of Pediatric Surgery, Juntendo University Urayasu Hospital, Urayasu, Chiba, Japan; ^6^ Department of Pediatrics and Adolescent Medicine, Juntendo University Graduate School of Medicine, Tokyo, Japan

**Keywords:** *P. lymphophilum* and monosymptomatic nocturnal enuresis category: original research article children, nocturnal enuresis, *Propionimicrobium lymphophilum*, urinary microbiome, urobiome

## Abstract

**Background:**

Despite a unique microbiome in urine, the relationship between nocturnal enuresis and the urobiome remains unclear. This study aimed to compare the presence of specific bacterial species in the urine of children with and without nocturnal enuresis.

**Methods:**

We used 16S ribosomal RNA gene sequencing to analyze the urobiome in urine samples obtained from the two groups of children. The presence of *Propionimicrobium lymphophilum* was examined using real-time PCR in the urine of 25 children diagnosed with monosymptomatic nocturnal enuresis (MNE), and 17 children without this condition.

**Results:**

Children with MNE exhibited a significantly higher prevalence of *P. lymphophilum*: 16 out of 25 (64.0%) compared to 4 out of 17 (23.5%) in the control group. Among children with frequent bedwetting, there was a significantly higher prevalence of *P. lymphophilum*;15 out of 16 (93.8%) compared to 2 out of 9 (22.2%) in those with infrequent bedwetting. Bacterial culture tests confirmed the anaerobic growth of *P. lymphophilum* isolates from urine samples of two PCR-positive patients with MNE. These isolates were found to be susceptible to ampicillin.

**Conclusion:**

These findings suggest that *P. lymphophilum* may be associated with chronic urinary tract infections and potentially contribute to the development of MNE in children.

## 1 Introduction

Nocturnal enuresis (NE), also known as bedwetting, is a common health issue affecting approx., 5% to 10% at 7 years, and 1% to 2% of adolescents (Bogaert et al., 2020). The International Children’s Continence Society (ICCS) defines NE as the unintentional release of urine during sleep at least once a month for at least three consecutive months in children over the age of 5 years (Austin et al., 2006; Nevéus et al., 2006). Although not life-threatening, bedwetting diminishes the quality of life of affected children and causes considerable stress for parents and children (Sarici et al., 2016). The current understanding suggests that high arousal thresholds, along with nocturnal polyuria or detrusor overactivity, contribute to the development of enuresis (Nevéus, 2017). However, the underlying factors associated with detrusor overactivity and inability to wake up during sleep remain unknown.

The microbiome refers to the microorganisms present in a specific environment (Gilbert et al., 2018). Research on the urinary microbiome, also known as the urobiome, has the potential to greatly enhance our understanding of genitourinary conditions, and may lead to the discovery of new treatments. Over the past decade, the relationship between microbes and humans has garnered much interest from researchers who have utilized next-generation sequencing (NGS) technologies. NGS-based analysis, specifically using bacterial 16S rRNA, has been widely used to identify clinical pathogens in urology (Wojciuk et al., 2019; Brubaker et al., 2021a; Brubaker et al., 2021b). Despite being considered sterile in traditional culture-based tests, urine harbors a distinct microbiome (Whiteside et al., 2015). The urobiome appears to play a role in the development of urological disorders, such as lower urinary tract symptoms (LUTS), interstitial cystitis, nephrolithiasis, and malignant neoplasms. However, research on the urobiome in children is limited, as the underlying conditions may differ from those observed in adults (Cole et al., 2022; Storm et al., 2022).

Recent studies on the urobiome in children have indicated that its composition changes during childhood and that there are differences in the bacterial flora between males and females (Storm et al., 2022). However, there is a lack of research comparing the urobiome of children with and without the disease, and no clinical investigations have explored the relationship between the urobiome and NE. Therefore, we hypothesized that there are different types of urobiome in the urine of children and that specific microorganisms may be responsible for causing NE. This study aimed to identify specific microorganisms that differ in the urobiome of children with and without primary monosymptomatic NE (MNE). Additionally, we aimed to determine whether specific bacterial species were present in the urine of patients with MNE.

## 2 Materials and methods

### 2.1 Ethics approval and consent

All study procedures, including urine sample collection, storage, and analysis, were approved by the ethics committee of the Juntendo University School of Medicine (No. U21-0014) and was in accordance with the tenets of the 1964 Declaration of Helsinki and its later amendments or comparable ethical standards. All parents and guardians provided written informed consent before inclusion in the study.

### 2.2 Participants and eligibility criteria

We enrolled 25 children (16 males; aged ≥5, mean 7.6 yrs.) with a diagnosis of primary MNE from the pediatric department of Juntendo University Urayasu Hospital, from whom urine samples were collected between June 2021 and May 2023. These patients were treatment-naïve to ensure that they had not received any prior treatment that might have affected the urobiome or confounded the results. Primary MNE is defined as one or more bedwetting episodes per month for a duration of at least three months in children aged ≥5 years based on the ICCS definition (Nevéus et al., 2006). The severity of bedwetting was categorized as frequent (≥ 4 times per week) or infrequent (<4 times per week), based on the ICCS definition (Austin et al., 2006).

The control group included 17 children (11 males; aged ≥5, mean 7.0 yrs.) who did not have NE and were undergoing or scheduled for minimally invasive surgery at the Pediatric Surgery Department of the Juntendo University Urayasu Hospital. The surgeries performed in the control group were inguinal hernias (8 cases), retractile testes (6 cases), scrotal hydrops (2 cases), and phimosis (1 case). All participants in the control group underwent abdominal ultrasound and urinalysis screening before inclusion in the study.

The exclusion criteria for participants in this study were as follows: individuals with symptoms of urinary tract infection such as fever or bladder irritation, or who have had a urinary tract infection (UTI) in the past 6 months, individuals who had taken antibiotics within the past six months, individuals with psychiatric disorders and behavioral disorders, including intellectual disability, attention-deficit/hyperactivity disorders, and individuals with constipation. The intellectual and behavioral problems were evaluated by a pediatric neurologist in accordance with developmental quotient (DQ)<75 according to Kyoto Scale of Psychological Development test, and Diagnostic and Statistical Manual of Mental Disorders fifth edition (DSM-V) consensus criteria were used for ADHD diagnosis (American Psychiatric Association, 2013; Kono et al., 2016). Constipation also was determined according to the ROME IV diagnostic criteria (Hyams et al., 2016).

Clinical data were collected from electronic medical records. The following baseline characteristics were recorded for all participants: sex, age, height, weight, body mass index, history of urinary tract infection, age at daytime diaper discontinuation, presence or absence of psychological or neurological pathologies, and presence or absence of constipation. In addition, we evaluated the severity of bedwetting on a weekly basis by using bladder diaries for participants in the MNE group.

### 2.3 Sample collection, DNA extraction, bacterial DNA amplification and real-time PCR

We used the commonly used midstream void method to collect urine samples from sterile cups (May, 2018). Pediatric nurse specialists disinfected the skin around the genital area and urethral meatus using 10% povidone-iodine to minimize contamination. After disinfection, midstream void urine specimens (30 mL) were collected and frozen at -80°C within 60 min of collection until batch processing. In the control group, we collected urine samples through transurethral catheterization in the operating room before administering preoperative antibiotics, in addition to clean-catch samples. For DNA isolation, 30 mL urine samples were centrifuged at 1,000 × g to form pellets. The pellets were resuspended in a total volume of 500 μl of phosphate-buffered saline. DNA was isolated from the urine samples using Power Fecal DNA Isolation Kits (QIAGEN, Venlo, the Netherlands). Urine samples were digested in a cocktail of lysozyme (10 mg/mL) and mutanolysin (25 KU/mL) for 1 h at 37°C. Next, 20% SDS and phenol: chloroform: isoamyl alcohol (25:24:1) were added and the samples were homogenized by bead beating with Mini-Beadbeater. DNA was precipitated and finally resuspended in 50 μl of DNA-free water.

For 16S rRNA gene sequencing, DNA libraries targeting the V3-V4 regions of the16S rRNA genes were prepared according to the Illumina 16S Metagenomic Sequencing Library Preparation Guide. The libraries were sequenced using MiSeq systems (Illumina, San Diego, CA, USA). To analyze the sequences, the 16S-based microbiome taxonomic profiling platform of EzBioCloud Apps and analyzing system was utilized (https://www.ezbiocloud.net/resources/16s_download).

Quantification of *P. lymphophilum* 16S rRNA by qPCR was performed using Thunderbird Probe qPCR Mix (TOYOBO, Japan) following the manufacturer’s instructions (Instruction manual THUNDERBIRD Probe qPCR Mix 2004. chrome-extension://efaidnbmnnnibpcajpcglclefindmkaj/ https://www.toyobo-global.com/seihin/xr/lifescience/support/manual/QPS-101.pdf.). The PCR mixture consisted of 10.0 µl of Thunderbird Probe qPCR Mix, 0.6 µl each of P. lymphophilum 16S rRNA-F (5’–3’) and *P. lymphophilum* 16S rRNA-R (5’–3’) primers (final concentration of 0.3 µmol each), 0.4 µL of *P. lymphophilum* 16S rRNA-Probe (5’-FAM- GTAGGGTGCGAGCGTTGTCC-BHQ1-3’, final concentration of 0.2 µmol), 0.4 µl of 50 x ROX reference dye, and 1.0 µL of extracted DNA (1mg/mL). The total volume of the mixture was adjusted to 20 µL with water. The abundance of *P. lymphophilum* 16S rRNA in each sample was quantified using a QuantStudio 3 real-time PCR system (Applied Biosystems). The qPCR protocol involved an initial heat denaturation step at 95°C for 60 s, followed by 45 cycles of denaturization at 95°C for 15 s, annealing at 60°C for 60 s, and extension.

### 2.4 Isolation and culture of *P. lymphophilum* and antimicrobial susceptibility test

To determine the antimicrobial susceptibility of *P. lymphophilum*, including a reference strain (accession no. JCM 5829) and two strains obtained from urine samples, the E-strip antimicrobial susceptibility method was employed. The MIC (minimum inhibitory concentrations) values for Ampicillin, Metronidazole and Trimethoprim-Sulfamethoxazole (bioMérieux, Marcy l’Etoile, France) were tested using the E-strips. The isolate was inoculated in GAM bouillon (Nissui, Tokyo, Japan) at 37°C for 2 days. Then the dried surface of a KBM Anaero RS-GNR plate was swabbed with the cultivated isolate using a sterile swab. Using forceps, an E-strip was placed on the surface of the plate. The plate was incubated under anaerobic conditions at 37°C for 24 h. And the antimicrobial susceptibility was assessed based on the resulting zone of inhibition.

### 2.5 Statistical analyses

The results are presented as mean and standard deviation (SD) for continuous variables and as counts (%) for categorical variables, unless otherwise indicated. Fisher’s exact test was used to compare categorical variables between the groups, and the Mann-Whitney U-test was used for continuous variables. Statistical analyses were performed using Statcel 4 software (OMS Publishing Inc., Saitama, Japan), with p < 0.05 deemed statistically significant.

## 3 Results

### 3.1 Baseline characteristics

Forty-two children participated in the study. The MNE group comprised of 25 children with MNE. The control group comprised 17 children without NE. There was no significant difference between the groups regarding sex, age, BMI, UTI history, or age in months when the children stopped using diapers during the daytime, indicating that daytime urinary continence was established. In the MNE group, 16 patients (64.0%) were classified into the frequent bedwetting group and 9 (36.0%) into the non-frequent bedwetting group (**Table 1**).

**Table 1:** Baseline characteristics of participants.

	Overall	MNE Group	Control Group	p value
No. of participants	42	25	17	
No. of male (%)	27 (64.3)	16 (64.0)	11 (64.7)	0.612
Mean SD age (yrs)	7.3 ± 1.4	7.6 ± 1.3	7.0 ± 1.4	0.060
Mean SD BMI (kg/m^2^)	17.1 ± 3.2	17.0 ± 3.3	15.9 ± 2.7	0.682
No. of history of UTI	0 (0)	0 (0)	0 (0)	0.588
Age out of diapers during the daytime (mos)	39.6 ± 5.2	40.4 ± 5.4	36.0 ± 4.4	0.276
Frequency of bedwetting (%)				
4 times or more/wk		16 (64.0)	0 (0)	
3 times or less/wk		9 (36.0)	0 (0)	

SD, standard deviation; BMI, body mass index; UTI, urinary tract infection.

### 3.2 Urobiome profiles

As a pilot study, we analyzed urine samples from two children with MNE (mean age 7.0 years; one male) and two children without NE (mean age 7.5 years; two males), though gender was not matched at the time of the pilot study. The urobiome profiles of each pair of children, with and without NE are illustrated in online **Supplementary Figure S1**. At the genus-level, the microbiota in the four urine samples consisted of 52 to 72 genera, with the most abundant being *Enterococcus*, followed by *Clostridium*, *Eubacterium* and *Bacillus* in order of abundance in all samples, with no significant differences in the percentages of these genera between the two NE and non-NE children. In addition, both pairs showed bacterial diversity in their urine samples, consisting of over 130 genera of bacterial species. There were no significant differences in α-diversity, Chao1, observed operational taxonomic units and Shannon, between the two groups. However, in samples from children with MNE, we detected the presence of a specific bacterial species, *P. lymphophilum*, as well as two bacterial families, *Fenollaria* and *Lachnospiraceae*. These were not detected in the urine samples from children without NE. We further confirmed the presence of *P. lymphophilum* using organism-specific real-time PCR analysis, which yielded positive results for samples from children with MNE. However, PCR targeting these two bacterial families has not been performed. Therefore, our focus was primarily on the presence or absence of *P. lymphophilum* in the urine samples from children with MNE.

### 3.3 Real-time PCR analysis of *P. lymphophilum* in urine of children with MNE

**Figure 1:**
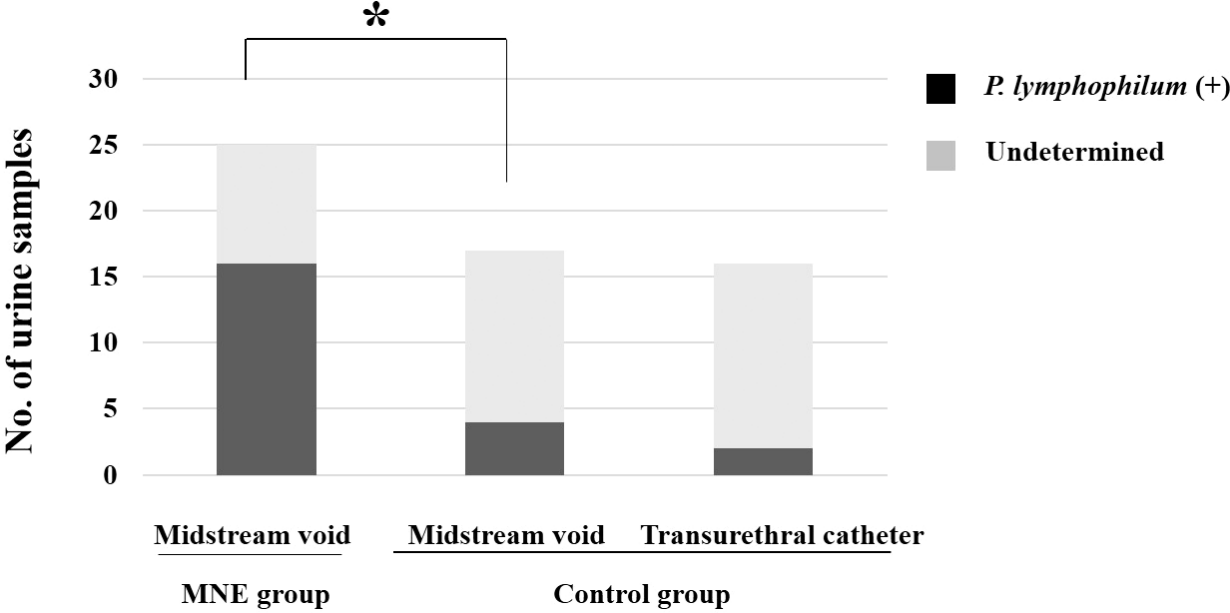
The graph illustrates the number of children from whom urine (samples) was obtained, and P. lymphophilum was identified using real-time PCR analysis. The asterisk indicates P. lymphophilum was detected in significantly (p <0.01) more children in the MNE group than in the control group. The two collection methods did not differ in detection rates in urine samples from the control group. MNE, monosymptomatic nocturnal enuresis; P. lymphophilum, *Propionimicrobium lymphophilum*.

The MNE group had a significantly higher rate of positive results for *P. lymphophilum* in urine than the control group. Of the 25 samples from the MNE group, 16 (64.0%) tested positive for *P. lymphophilum* by PCR, whereas only 4 out of 17 from the control group (23.5%) were PCR-positive (p <0.001, **Figure 1**).

The MNE group only used clean-catch midstream urine samples. On the other hand, the control group used two different methods to collect urine samples: clean-catch midstream urine and transurethral catheterization during surgery. There was no difference in the DNA of *P. lymphophilum* identified in the urine samples collected using the two methods in the control group.

### 3.4 Real-time PCR-positivity of *P. lymphophilum* in urine and bedwetting severity

Patients with severe MNE with ≥4 bedwetting/week showed a significantly higher positive rate of *P. lymphophilum* in urine than patients with non-severe MNE with ≤ 3times bedwetting/week. Specifically, 15 out of 16 patients (94%) with severe MNE were PCR-positive for *P. lymphophilum*, while only two out of nine (22%) from the control group were PCR-positive (**Table 2**).

**Table 2:** Comparison of *P. lymphophilum* positivity by bedwetting frequency in MNE groups.

	Frequency of bedwetting/wk		p value
	4 times or more (n=16)	3 times or less (n=9)	
No. of positive sample (%)	15 (93.8)	2 (22.2)	<0.001
No. of negative sample (%)	1 (6.2)	7 (77.8)

MNE, monosymptomatic nocturnal enuresis.

### 3.5 Urine culture and antimicrobial susceptibility of *P. lymphophilum*

**Figure 2:**
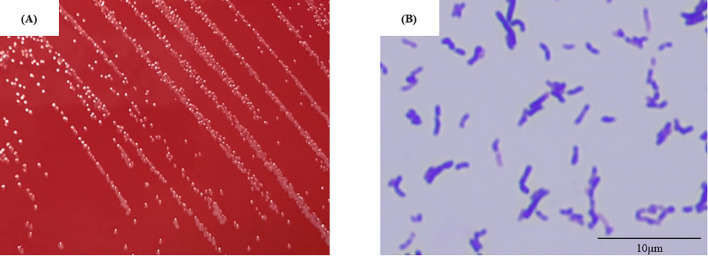
Isolate No.7 **(A)** Colony appearance after four days of anaerobic incubation on selective medium. The colonies were circular, light grey to white, and raised or convex with entire margins. **(B)** Gram-positive bacilli are observed in the gram stain (showing pleomorphism, including club- shaped and coccoid forms). (Original magnification, × 1,000).

To identify *P. lymphophilum* in children with MNE, urine samples from two individuals (No. 7 and No. 11; **Supplementary Table 1**) was used to culture *P. lymphophilum* in selective medium. Gram staining revealed the presence of Gram-positive bacilli (**Figure 2**). The 16S rRNA sequences of these strains were compared with the 16S rRNA sequence of the reference strain *P. lymphophilum* (accession no. JCM 5829). Based on this comparison, we confirmed that the isolates grown on culture plates were *P. lymphophilum*.

Furthermore, the minimal inhibitory concentration (MIC) of the *P. lymphophilum* strains to various antibiotics was assessed. The strains were found to be susceptible to ampicillin (MIC = 0.47 mg/L), However, they were considered resistant to metronidazole (MIC > 256 mg/L), and trimethoprim-sulfamethoxazole (MIC > 32 mg/L) (**Table 3**).

**Table 3:** Antimicrobial susceptibilities by E-test.

	MIC(µg/mL)		
	*P. lymphophilum* (accession no. JCM 5829)	*P. lymphophilum* from No.7 participant	*P. lymphophilum* from No.11 participant
Ampicillin	0.23	0.47	0.94
Metronidazole	>32	>32	>32
Trimethiprim-Sulfamethoxazole	>256	>256	>256

MIC, minimal inhibitory concentration.

## 4 Discussion

The urobiome, which refers to the microbial community present in the urinary tract, exists in both children and adults. Siddiqui et al. were the first to report in 2011 that multiple DNAs of bacterial origin are present in the urine of healthy women, even when culture tests are negative, forming a bacterial flora (Siddiqui et al., 2011). Since then, urobiome and various pathophysiologic conditions also have been reported in children. A recent review article (Cole et al., 2022) has summarized recent research findings on the urobiome in children, highlighting the potential to identify novel therapeutic approaches. Studies on the urobiome in children have investigated both healthy children and those with diseases, including neuropathic bladder, UTI, nephrolithiasis, and bladder and bowel dysfunction (Cole et al., 2022; Cole et al., 2023; Forster C et al., 2023); however, there were no studies on the relationship between NE and UTI in urobiome research. our research confirmed that the urobiome in children exhibits diversity, consisting of 130 genera, which is consistent with a previous report (Whiteside et al., 2015). The presence of dysbiosis or specific microorganisms in the urine can potentially contribute to the development of diseases in children, such as MNE.

The pathogenicity of *P. lymphophilum* in children is not well understood. However, several reports have indicated that *P. lymphophilum* is a causative agent of UTI in adults (Williams, 2015; Ikeda et al., 2017; Cobo et al., 2021; Fernández Vecilla et al., 2023). It has also been isolated from prostate cancer (Shrestha et al., 2018). *P. lymphophilum* is classified as an anaerobic Gram-positive bacillus and is known to exist in the human skin and urinary tract in adults. Its presence was initially reported by Torrey in 1916, who referred to the bacteria isolated from lymph nodes of Hodgkin’s disease patients as “Bacillus lymphophilus” (Torrey, 1916). In 2002, the genus *Propionimicrobium* was proposed to belong to the family *Propionibacteriaceae*, with *P. lymphophilum* being the sole species identified within this genus (Stackebrandt et al., 2002). Although *P. lymphophilum* is a component of some microbiomes, its association with pathological disorders is evident under some conditions.

It is possible that *P. lymphophilum* may directly stimulate the bladder mucosa in children with MNE since this microbe has been reported to produce propionic acid. An experimental study demonstrated that short-chain fatty acids, including propionic acid, play a role in stimulating sodium transport during contraction of the bladder mucosa in toads (Hess et al., 1975). Additionally, the calcium signaling pathway, along with adenosine triphosphate-mediated pathways involving sodium and potassium, are important for bladder contraction, contributing to overactive bladder (OAB) conditions (Olsen et al., 2011; Birder and Andersson, 2013; Merrill et al., 2016). Considering these findings, it is possible that the presence of *P. lymphophilum* in the urine of children with MNE may be associated with propionate-mediated OAB, which could potentially contribute to bedwetting.

Another potential explanation for *P. lymphophilum* being a causative agent of MNE among children is that this microbe or its products may inhibit arousal associated with the need to urinate. A recent study emphasized the association between the urobiome composition and the central nervous system in adults (Wu et al., 2017). Changes in the diversity of bacteria in urine have been found to lead to psychological conditions in adult females with OAB. It is possible that bacteria in urine produce certain neurotransmitters that interact with the nervous system (Whiteside et al., 2015). Based on these findings, the presence of *P. lymphophilum* in urine may play a role in sleep, including the inability to awaken with urine in children with MNE. Chemotherapy, including the use of ampicillin, is considered a therapeutic approach for patients with MNE who test positive for *P. lymphophilum* in urine. Ampicillin is a cost-effective and safe antibacterial agent that is commonly prescribed for children with tonsillitis and other upper respiratory tract infections. Although 16S rRNA analysis was used to detect DNA from dead bacteria in the urine, this study successfully cultured live bacteria of *P. lymphophilum* from urine samples of patients with MNE, indicating its presence. The isolated bacteria were found to be susceptible to ampicillin. While our institution is currently developing treatment protocols and continuing IRB review, we still need to build on our knowledge of MNE and urobiome, such as in this study.

The current study had several limitations. First, it included a small number of patients with MNE from a single institution. This is due to the difficulty in recruiting patients and obtaining their consent to participate in the study, as many patients with uncomplicated MNE do not make their first visit to our hospital in an untreated state. To overcome these difficulties, a multicenter study is currently under consideration. Second, except for *P. lymphophilum*, two bacterial families *Fenollaria* and *Lachnospiraceae* was detected in urine samples from NE patients (**Supplementary Figure S1**), and they may contain causative bacterial species of NE. Further study would be necessary to detect these species. Third, the MNE group used the midstream void clean-catch method for urine sample collection. Although urine samples have a low biomass and are prone to contamination and variability depending on the collection methods (Merrill et al., 2016), the clean-catch method is suitable for non-life-threatening conditions such as NE and can be easily performed in clinical settings. While the choice between clean-catch and catheterization methods for urine collection in adult patients is controversial (Eisenhofer et al., 2019; Pohl et al., 2020; Ozer et al., 2021), neither method significantly affected the detection of *P. lymphophilum* in urine samples in the control group in this study. Fourth, the study did not analyze patients with MNE according to age. Although the participants in this study were mainly around seven years of age, which is the most prevalent age for NE, future research should investigate the urobiome in older children, including menstruating and post-pubertal patients, as certain physiological changes may impact the urobiome.

Despite these limitations, we should highlight that *P. lymphophilum* is a potential cause or likely involved in the condition of patients with MNE. Future prospective studies with larger sample sizes are needed to strengthen the reliability of our findings.

## 5 Conclusions

In conclusion, this is the first study to report the urobiome of children with NE. These results suggest that chronic UTI caused by *P. lymphophilum* may be one of possible cause of MNE in children. If antimicrobial therapy was effective for MNE, it would be the new impact on the practice of NE treatment in addition to alarm therapy or desmopressin acetate. Further prospective studies are necessary to confirm the robustness of these results.

## Data Availability

The 16S metagenomic sequencing data have been deposited at the GenBank/EMBL/DDBJ database under accession number DRR303664 to DRR303669.
