# Supplementary material for: Propionimicrobium lymphophilum in urine of children with monosymptomatic nocturnal enuresis
Source: Front Cell Infect Microbiol. 2024 Nov 25;14:1377992. doi: 10.3389/fcimb.2024.1377992 (PMC11626389; doi:10.3389/fcimb.2024.1377992)
Supplement: Supplementary file 1 [file SupplementaryFile1.docx]

Supplemental Figure 1


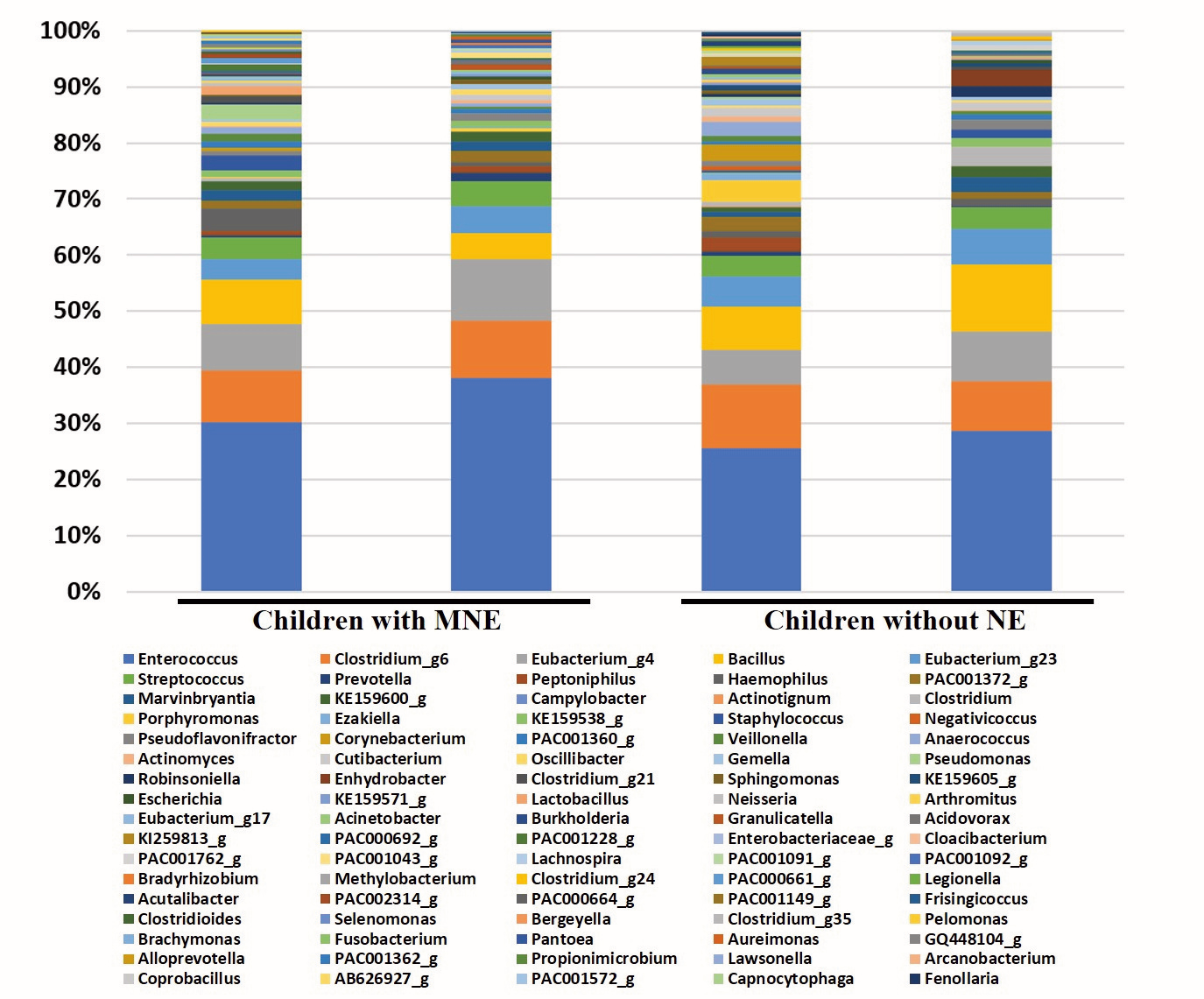


Relative abundance of taxa at the order level in children with MNE compared to those without NE. The heights of the color bars represent the percentage of each taxon. MNE, monosymptomatic nocturnal enuresis; NE, nocturnal enuresis.
