# Supplementary material for: Propionimicrobium lymphophilum in urine of children with monosymptomatic nocturnal enuresis
Source: Front Cell Infect Microbiol. 2024 Nov 25;14:1377992. doi: 10.3389/fcimb.2024.1377992 (PMC11626389; doi:10.3389/fcimb.2024.1377992)
Supplement: Supplementary file 2 [file Table1.docx]

Supplemental Table 1

Comparative analysis of *Ct* values for real-time PCR from clinical samples of MNE and the control group

MNE group

participants No.	Age (yr)	Gender	Threshold cycle (Ct) values	Bedwetting frequency/wk (number)
1	9	F	40.567	7
2	6	M	40.460	7
3	8	M	0	3
4	6	M	0	2
5	9	M	35.930	5
6	7	M	39.668	7
7	8	F	36.942	5
8	6	M	39.906	7
9	9	F	37.993	6
10	8	M	43.763	7
11	6	F	34.493	6
12	7	M	0	3
13	8	F	0	3
14	9	F	40.328	7
15	5	M	38.984	7
16	9	M	40.498	7
17	9	M	0	2
18	9	M	0	2
19	8	F	35.208	7
20	8	M	0	7
21	8	M	33.798	7
22	5	M	42.228	5
23	7	M	0	7
24	9	F	0	3
25	8	F	39.370	2

Control group

participants No.	Age (yr)	Gender	Threshold cycle (Ct) values	Bedwetting frequency/wk (number)
1	8	M	0	none
2	9	M	32.807	
3	8	M	32.486	
4	8	F	0	
5	5	M	34.853	
6	8	M	0	
7	8	M	0	
8	5	F	0	
9	6	F	0	
10	8	F	0	
11	5	M	33.945	
12	5	F	0	
13	5	M	0	
14	7	F	0	
15	7	M	0	
16	7	M	0	
17	7	M	0	
